# Incidence of left ventricular thrombus and subclinical cerebral infarction by magnetic resonance imaging 2 weeks after anterior ST-elevation myocardial infarction with impaired left ventricular function and apical akinesis

**DOI:** 10.1093/ehjci/jeag267

**Published:** 2026-09-25

**Authors:** Thomas Anderton, Lisa Borg, Moosa Shaikh, Rabeia Javid, Raluca Tomoaia, Salman Arain, Stuart Currie, Russell Frood, Heerajnarain Bulluck, Sven Plein

**Affiliations:** Leeds Institute for Cardiovascular and Metabolic Medicine, University of Leeds, 6 Clarendon Way, Leeds LS2 9JT, United Kingdom; Leeds Institute for Cardiovascular and Metabolic Medicine, University of Leeds, 6 Clarendon Way, Leeds LS2 9JT, United Kingdom; Leeds Institute for Cardiovascular and Metabolic Medicine, University of Leeds, 6 Clarendon Way, Leeds LS2 9JT, United Kingdom; Leeds Institute for Cardiovascular and Metabolic Medicine, University of Leeds, 6 Clarendon Way, Leeds LS2 9JT, United Kingdom; Department of Cardiology, Iuliu Hațieganu University of Medicine and Pharmacy, Cluj-Napoca, Romania; Department of Neuroradiology, Leeds General Infirmary, Great George Street, Leeds, UK; Department of Neuroradiology, Leeds General Infirmary, Great George Street, Leeds, UK; Leeds Institute of Clinical Trials Research, University of Leeds, Woodhouse Lane, Leeds, UK; Leeds Institute for Cardiovascular and Metabolic Medicine, University of Leeds, 6 Clarendon Way, Leeds LS2 9JT, United Kingdom; Leeds Institute for Cardiovascular and Metabolic Medicine, University of Leeds, 6 Clarendon Way, Leeds LS2 9JT, United Kingdom

**Keywords:** LV thrombus, STEMI, CMR

## Abstract

**Graphical Abstract:**
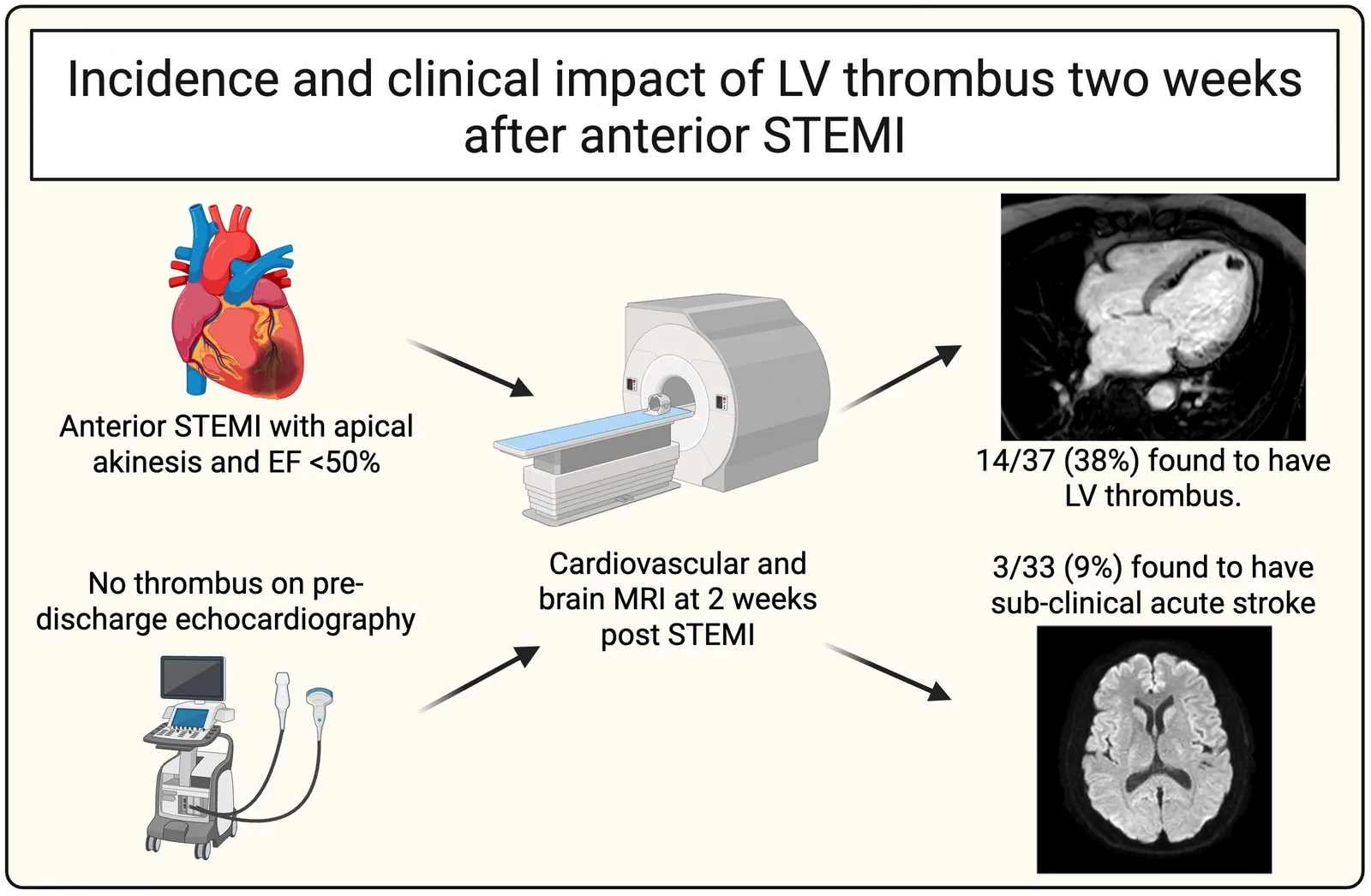

Patients with anterior ST-elevation myocardial infarction (STEMI), apical akinesia, and left ventricular ejection fraction (LV EF) <50% are at high risk for LV thrombus formation,^1^ with clinical guidelines recommending the exclusion of LV thrombus with a pre-discharge echocardiogram in this patient group, regardless of individual risk.^2^ However, previous studies suggest that the incidence of LV thrombus peaks much later at around 2 weeks post-MI and may not be detected by pre-discharge echocardiography screening.^1^ We sought to determine the incidence of LV thrombus at 14 days after infarct in high-risk patients post-STEMI without LV thrombus on pre-discharge echocardiography. We used cardiovascular magnetic resonance (CMR) as the most sensitive test to detect LV thrombus, and brain magnetic resonance imaging (MRI) to detect acute cerebral ischaemia as a potential sequela of LV thrombus formation.

This was a prospective and pre-specified study. Consecutive patients presenting to our centre between January 2024 and January 2026 with anterior STEMI, LV EF <50%, and apical akinesia but no LV thrombus on a pre-discharge echocardiogram were approached. Non-contrast echocardiography was performed 12–48 h after acute admission by British Society of Echocardiography–accredited sonographers within the coronary care unit as per local practice and in accordance with practice guidelines for post-STEMI care. Exclusion criteria included absolute contraindications to CMR/MRI and pre-existing reasons for long-term anticoagulation. All patients were prescribed aspirin and prasugrel on discharge, with none prescribed anticoagulation either at discharge or between discharge and CMR.

Patients underwent CMR/MRI on a Philips Ingenia 1.5T (Philips Healthcare, the Netherlands) scanner 14 days after STEMI. The CMR protocol included cine imaging and early and late gadolinium enhancement. Post-processing and analysis were performed using cvi42 (version 6.12, Circle Cardiovascular Imaging, Calgary, Canada). LV thrombus was defined as an intracavity lesion with low signal intensity surrounded by blood pool visible on both early and late gadolinium-enhanced images. It was carefully distinguished from microvascular obstruction (MVO), which was defined as a low-signal intramyocardial lesion completely surrounded by hyperenhanced myocardium visible on early gadolinium-enhanced images with similar or reduced size on late gadolinium enhancement. Cranial imaging consisted of T2-weighted turbo-spin echo, T2-weighted fast field gradient echo, and diffusion-weighted imaging.

For comparison, patients were grouped into those with and without thrombus. Independent sample *t* test and Mann–Whitney *U* test were used as appropriate, with normally distributed variables presented as means ± standard deviation and non-normal as median (interquartile range). Proportions were compared with χ^2^ squared. *P* values of <0.05 were considered significant. The study was approved by the research ethics committee (18/YH/0168). All participants signed written informed consent.

Of the patients admitted with anterior STEMI in the recruitment period, 45 met the inclusion criteria and agreed to participate with 37 subsequently completing CMR (six did not attend and two were claustrophobic). Of these, 14 (38%) had CMR evidence of LV thrombus on the 14-day CMR scan. Among patients with LV thrombus, 12 of 14 (86%) had a single LV thrombus and two (14%) had multiple. The median size of LV thrombus per positive case was 57 mm^2^ [35–120 mm^2^]. Eleven (79%) patients were described as protruding into the LV cavity, with the remainder laminar and no thrombi were reported as mobile. Those with LV thrombus were younger (52 vs. 63 years, *P* = 0.015), had higher incidence of co-existent microvascular obstruction (MVO) (79% vs. 35%, *P* = 0.011), and had higher incidence of persistent apical akinesis at CMR (93% vs. 52%, *P* = 0.011). All patients with confirmed LV thrombus on day-14 CMR had their prescription changed to clopidogrel and apixaban per local practice.

Of 37 participants, 33 completed cranial MRI (4 did not tolerate the head coil). None of these patients had symptoms or signs of stroke. Three patients (3/33, 9%) had radiological evidence of acute brain infarction. Given the small numbers, no meaningful difference between groups could be determined. *Table 1* compares the demographics, CMR and brain MR findings of those with and without LV thrombus.

In this study, contrast-enhanced CMR detected LV thrombus at 14 days in over one-third of patients with high-risk STEMI who had no evidence of thrombus on standard-of-care pre-discharge echocardiography. This observation raises important questions about the optimal timing and modality used for pre-discharge screening for LV thrombus post STEMI.

The observed incidence of LV thrombus in our cohort was higher than that reported in most previous studies. Two previous post-STEMI studies have reported thrombus incidences of 8.6% and 4%^3,4^ at 30 and 7 days, respectively. Both previous studies used predominantly echocardiography and did not require the presence of apical akinesis, thereby capturing a lower risk population than the current report and utilizing a less sensitive imaging modality.

Our study also aimed to determine if the presence of LV thrombus is associated with clinically significant embolic events. Three of the 33 patients in this study had clinically silent stroke on MRI at 2 weeks, but there was no difference in stroke incidence or cerebral microvascular changes between patients with and without LV thrombus. These numbers are too small to draw conclusions on the prognostic relevance of undetected LV thrombus, and larger studies with longer follow-up will be required.

This study has important limitations. Echocardiography and CMR were not performed at the same time points by study design, preventing comparisons between the detection rate of both modalities at different time points. This study cannot therefore distinguish between patients who had LV thrombus pre-discharge that was missed by echocardiography and those who developed LV thrombus subsequently. The rationale for this study design was to compare current guideline-directed care with the assumed optimal imaging modality by CMR and timing at 2 weeks post-MI.

While preliminary, this study indicates that the incidence of LV thrombus at 2 weeks in these high-risk patients is high, even if pre-discharge echocardiography is negative, and informs future studies aimed at clarifying whether the highest-risk group may benefit from routine CMR. While pre-discharge CMR is a possibility, it may miss later developing thrombi and may be less feasible in most hospitals than an outpatient scan after 2 weeks. These observations call for larger prospective studies with standardized echocardiography, quantitative CMR phenotyping, and clinical outcome follow-up. Such studies could inform not only the optimal timing of LV thrombus screening post-MI and the optimal screening method but also the potential value of prophylactic or imaging-guided anticoagulation.

**Table 1:** Table comparing demographics, CMR and brain MR findings of those with and without LV thrombus

	Confirmed LV thrombus *N* = 14	No LV thrombus *N* = 23	*P* value	All *N* = 37
Demographics				
Age (years)	52 (±11)	63 (±11)	0.015	59 (±13)
Male (*n*)	12 (86%)	17 (74%)	0.404	29 (78%)
Caucasian (*n*)	8 (57%)	19 (83%)	0.095	27 (73%)
BMI (kg/m^2^)	27.0 (±6.7)	27.5 (±4.7)	0.767	27 (±5)
Hypertension (*n*)	5 (36%)	7 (30%)	0.743	12 (32%)
Diabetes mellitus (*n*)	2 (14%)	6 (26%)	0.404	8 (22%)
Current/past smoker (*n*)	9 (65%)	9 (39%)	0.143	18 (49%)
Time-to-wire (hours)	4.75 (2.50–10.25)	4.00 (2.25–4.50)	0.388	4.00 (2.25–6.25)
Pre-discharge echocardiogram				
LV EF (%)	34 (±8)	37 (±8)	0.238	36 (±8)
Apical akinesis (*n*)	14 (100%)	23 (100%)	—	37 (100%)
2-week CMR				
LV EDV/BSA (mL/m^2^)	94 (±20)	84 (±18)	0.152	88 (±19)
LV Mass/BSA (g/m^2^)	118 (± 31)	114 (± 27)	0.720	115 (± 28)
LV EF (%)	43 (±8)	46 (±9)	0.319	44 (±9)
Apical akinesis (*n*)	13 (93%)	12 (52%)	0.011	25 (68%)
Segments of infarction (*n*)	7 (±1)	6 (±3)	0.365	7 (±3)
Infarction as % of LV mass (%)	41 [33–50]	41 [18–49]	0.707	41 [28–49]
MVO (*n*)	11 (79%)	8 (35%)	0.011	19 (51%)
MVO as % of LV mass (%)	2.2 [1.5–4.5]	2.2 [1.4–2.8]	0.562	2.2 [1.5–4.3]
Brain MRI	*n* = 11	*n* = 22		
Stroke (*n*)	1 (9%)	2 (9%)	1.00	3/33 (9%)
Fazekas score ≥1 (*n*)	3 (27%)	9 (41%)	0.437	12 (36%)

BMI, body mass index; BSA, body surface area; EDV, end diastolic volume; EF, ejection fraction.

## Data Availability

Study data will be made available upon reasonable request if made in writing to the corresponding author.
